# On the Set of the Numbers of Conjugates of Noncyclic Proper Subgroups of Finite Groups

**DOI:** 10.1155/2013/430870

**Published:** 2013-10-27

**Authors:** Jiangtao Shi, Cui Zhang

**Affiliations:** ^1^School of Mathematics and Information Science, Yantai University, Yantai 264005, China; ^2^Department of Applied Mathematics and Computer Science, Technical University of Denmark, 2800 Lyngby, Denmark

## Abstract

Let *G* be a finite group and *𝒩𝒞*(*G*) the set of the numbers of conjugates of noncyclic proper subgroups of *G*. We prove that (1) if |*𝒩𝒞*(*G*)| ≤ 2, then *G* is solvable, and (2) *G* is a nonsolvable group with |*𝒩𝒞*(*G*)| = 3 if and only if *G*≅*PSL*(2,5) or *PSL*(2,13) or *SL*(2,5) or *SL*(2,13).

## 1. Introduction

In this paper, all groups are assumed to be finite. It seems interesting to investigate the influence of some arithmetic properties of noncyclic proper subgroups on the solvability of groups. In [1], Li and Zhao proved that any group having at most three conjugacy classes of noncyclic proper subgroups is solvable, and a group *G* having exactly four conjugacy classes of noncyclic proper subgroups is nonsolvable if and only if *G*≅*PSL*(2,5) or *SL*(2,5). As a generalization of the above result, we showed that any group having at most three conjugacy classes of nonnormal noncyclic proper subgroups is solvable, and a group *G* having exactly four conjugacy classes of nonnormal noncyclic proper subgroups is nonsolvable if and only if *G*≅*PSL*(2,5) or *SL*(2,5) (see [2]).

Let *G* be a group and *𝒩𝒞*(*G*) the set of the numbers of conjugates of noncyclic proper subgroups of *G*. It is clear that a group *G* with *𝒩𝒞*(*G*) = *∅* is either a cyclic group or a minimal noncyclic group, and a group *G* with *𝒩𝒞*(*G*) = {1} is a group in which every noncyclic proper subgroup is normal. In [2], we also obtained a complete classification of groups *G* in which every noncyclic proper subgroup is nonnormal; all such groups *G* satisfy 1 ∉ *𝒩𝒞*(*G*).

By |*𝒩𝒞*(*G*)| we denote the order of *𝒩𝒞*(*G*). Note that we cannot ensure that 1 ∈ *𝒩𝒞*(*G*) for any solvable group *G* with |*𝒩𝒞*(*G*)| = *n* ≥ 1. For example, let *G*≅*D*_2*p*^*n*^_ be a dihedral group of order 2*p* ^*n*^, where *n* ≥ 1 and *p* is an odd prime. Then *𝒩𝒞*(*D*_2*p*^*n*^_) = {*p*, *p* ^2^,…, *p* ^*n*^}, so 1 ∉ *𝒩𝒞*(*D*_2*p*^*n*^_). For the nonsolvable group of the smallest order *PSL*(2,5), it is easy to see that *𝒩𝒞*(*PSL*(2,5)) = {5, 6, 10}, and so |*𝒩𝒞*(*PSL*(2,5))| = 3.

For the influence of |*𝒩𝒞*(*G*)| on the solvability of groups, we have the following result, the proof of which is given in Section 3.

**Theorem 1**

Let *G* be a group.

1. If |*𝒩𝒞*(*G*)|≤2, then *G* is solvable.
2. *G* is a nonsolvable group with |*𝒩𝒞*(*G*)| = 3 if and only if *G*≅*PSL*(2,5) or *PSL*(2,13) or *SL*(2,5) or *SL*(2,13).

The following two corollaries are direct consequences of Theorem 1.

**Corollary 2**

Let *G* be a group with |*𝒩𝒞*(*G*)|≤3. Then *G* is nonsolvable if and only if *𝒩𝒞*(*G*) = {5,6, 10} or {14,78,91}.

**Corollary 3**

Let *G* be a group and *𝒩𝒯*(*G*) the set of the numbers of conjugates of nontrivial subgroups of *G*.

1. If |*𝒩𝒯*(*G*)|≤2, then *G* is solvable.
2. *G* is a nonsolvable group with |*𝒩𝒯*(*G*)| = 3 if and only if *G*≅*PSL*(2,13).

Let *G* be a group and 𝒩𝒞*(*G*) the set of the numbers of conjugates of nonnormal noncyclic proper subgroups of *G*. Obviously 𝒩𝒞*(*G*)⊆*𝒩𝒞*(*G*).

Arguing as in the proof of Theorem 1, we can obtain the following result.

**Theorem 4**

Let *G* be a group. If |𝒩𝒞*(*G*)|≤2, then *G* is solvable.

**Remark 5**

If we assume that *G* is a nonsolvable group with |𝒩𝒞*(*G*)| = 3, we cannot get that Φ(*G*) = *Z*(*G*). For example, let *G*≅*PSL*(2,5) × *ℤ*_*p*_, where *p* ≥ 7 is a prime. It is easy to see that |𝒩𝒞*(*G*)| = 3. But Φ(*G*) = 1 and *Z*(*G*) = *ℤ*_*p*_.

Let *G* be a group and *𝒩𝒜*(*G*) the set of the numbers of conjugates of nonabelian proper subgroups of *G*. Obviously *𝒩𝒜*(*G*)⊆*𝒩𝒞*(*G*). Arguing as in the proof of Theorem 1, we can also obtain the following result.

**Theorem 6**

Let *G* be a group. If |*𝒩𝒜*(*G*)|≤2, then *G* is solvable.

## 2. Preliminaries

In this section, we collect some essential lemmas needed in the sequel.

**Lemma 7 (see [3])**

Let *G* be a group. If all nonnormal maximal subgroups of *G* have the same order, then *G* is solvable.

**Lemma 8 (see [4])**

Let *G* be a nonsolvable group having exactly two classes of nonnormal maximal subgroups of the same order; then *G*/*S*(*G*)≅*PSL*(2,7), where *S*(*G*) is the largest solvable normal subgroup of *G*.

**Lemma 9 (see [5, 6])**

Let *G* be a group having exactly *n* classes of maximal subgroups of the same order, where 1 ≤ *n* ≤ 3; then one of the following statements holds:

1. suppose that *G* is a group with *n* = 1, and then *G* is a *p*-group for some prime *p*;
2. suppose that *G* is a nonsolvable group with *n* = 2, and then *G*/Φ(*G*)≅(*ℤ*_2_^3*i*^⋊*PSL*(2,7)) × *ℤ*_7_^*j*^, where *i*, *j* = 0, 1,…, and *ℤ*_2_^3*i*^⋊*PSL*(2,7) is a semidirect product of the normal subgroup *ℤ*_2_^3*i*^ and the subgroup *PSL*(2,7);
3. suppose that *G* is a nonsolvable group with *n* = 3, and then *G*/*S*(*G*)≅*A*_6_; *PSL*(2, *q*), *q* = 11, 13, 23, 59, 61; *PSL*(3,3); *U*_3_(3); *PSL*(5,2); *PSL*(2, 2^*f*^), and *f* is a prime; *PSL*(2,7) × *PSL*(2,7) × ⋯×*PSL*(2,7).

## 3. Proof of Theorem 1

The proof of Theorem 1 follows from the following two lemmas.

**Lemma 10**

Let *G* be a group. If |*𝒩𝒞*(*G*)|≤2, then *G* is solvable.

**Proof**

Assume that *G* is nonsolvable. Then by [7, Exercise 10.5.7], all maximal subgroups of *G* are noncyclic. Let *ℳ𝒮*(*G*) be the set of the numbers of conjugates of maximal subgroups of *G*. It follows that *ℳ𝒮*(*G*)⊆*𝒩𝒞*(*G*). Then |*ℳ𝒮*(*G*)|≤2.

1. Suppose that 1 ∈ *ℳ𝒮*(*G*). Since *G* is nonsolvable, *G* must have nonnormal maximal subgroups. Let *M* be any nonnormal maximal subgroup of *G*; one has |*G* : *N*_*G*_(*M*)| = |*G* : *M*|. Since |*ℳ𝒮*(*G*)|≤2, we know that *G* has at most one class of nonnormal maximal subgroups of the same order. It follows that *G* is solvable by Lemma 7, a contradiction.
2. Suppose that 1 ∉ *ℳ𝒮*(*G*). It follows that all maximal subgroups of *G* are nonnormal. By the hypothesis, *G* has at most two classes of maximal subgroups of the same order. Since *G* is nonsolvable and *G* has no normal maximal subgroups, one has *G*/Φ(*G*)≅*ℤ*_2_^3*i*^⋊*PSL*(2,7) by Lemma 9 (1) and (2), where *i* = 0, 1,…. It is easy to see that *𝒩𝒞*(*G*/Φ(*G*))⊆*𝒩𝒞*(*G*) and |*𝒩𝒞*(*ℤ*_2_^3*i*^⋊  *PSL*(2,7))|>2. It follows that |*𝒩𝒞*(*G*)|>2, a contradiction.

Thus, our assumption is not true, so *G* is solvable.

**Lemma 11**

A group *G* is a nonsolvable group with |*𝒩𝒞*(*G*)| = 3 if and only if *G*≅*PSL*(2,5) or *PSL*(2,13) or *SL*(2,5) or *SL*(2,13).

**Proof**

The sufficiency part is evident, and we only need to prove the necessity part.

By the hypothesis, |*ℳ𝒮*(*G*)|≤3. We claim that

(1) $$\begin{array}{c} 1\notin ℳ𝒮\left(G\right). \end{array}$$

Otherwise, assume that 1 ∈ *ℳ𝒮*(*G*). Then *G* has at most two classes of nonnormal maximal subgroups of the same order. Since *G* is nonsolvable, one has *G*/*S*(*G*)≅*PSL*(2,7) by Lemmas 7 and 8. It is easy to see that *𝒩𝒞*(*G*/*S*(*G*))⊆*𝒩𝒞*(*G*) and |*𝒩𝒞*(*PSL*(2,7))|>3. It follows that |*𝒩𝒞*(*G*)|>3, a contradiction. Thus, 1 ∉ *ℳ𝒮*(*G*).

Since |*ℳ𝒮*(*G*)|≤3, we have that *G* has at most three classes of maximal subgroups of the same order.

By Lemma 9 (1), *G* cannot have exactly one class of maximal subgroups of the same order.

If *G* has exactly two classes of maximal subgroups of the same order, according toLemma 9 (2), one has *G*/Φ(*G*)≅*ℤ*_2_^3*i*^⋊*PSL*(2,7) since *G* has no normal maximal subgroups, where *i* = 0, 1,…. Since |*𝒩𝒞*(*ℤ*_2_^3*i*^⋊*PSL*(2,7))|>3, it follows that |*𝒩𝒞*(*G*)|>3, a contradiction.

Thus, *G* has exactly three classes of maximal subgroups of the same order. By Lemma 9 (3), *G*/*S*(*G*) might be isomorphic to *A*_6_ or *PSL*(2, *q*), *q* = 11, 13, 23, 59, 61 or *PSL*(3,3) or *U*_3_(3) or *PSL*(5,2) or *PSL*(2, 2^*f*^), and *f* is a prime or *PSL*(2,7) × *PSL*(2,7)×⋯×*PSL*(2,7). If *G*/*S*(*G*) is an isomorphism to *A*_6_ or *PSL*(2, *q*), *q* = 11, 23, 59, 61 or *PSL*(3,3) or *U*_3_(3) or *PSL*(5,2) or *PSL*(2, 2^*f*^), and *f* is an odd prime or *PSL*(2,7) × *PSL*(2,7) × ⋯×*PSL*(2,7). It is easy to see that |*𝒩𝒞*(*G*/*S*(*G*))|>3 by [8, 9], which implies that |*𝒩𝒞*(*G*)|>3, a contradiction. Thus, *G*/*S*(*G*)≅*PSL*(2,4)≅*PSL*(2,5) or *PSL*(2,13).

Note that 1 ∉ *ℳ𝒮*(*G*) and |*ℳ𝒮*(*G*)| = |*𝒩𝒞*(*G*)| = 3. It follows that 1 ∉ *𝒩𝒞*(*G*), so *S*(*G*) is cyclic. We claim that

(2) $$\begin{array}{c} \Phi \left(G\right)=S\left(G\right). \end{array}$$

Otherwise, assume that Φ(*G*) < *S*(*G*). Let *M* be a maximal subgroup of *G* such that *S*(*G*)≰*M*. Then *G* = *S*(*G*)*M*. It is obvious that *S*(*G*)∩*M*⊴*M*. Moreover, *S*(*G*)∩*M*⊴*S*(*G*), since *S*(*G*) is cyclic. It follows that *S*(*G*)∩*M*⊴*G*. Therefore, *G*/(*S*(*G*)∩*M*) = *S*(*G*)/(*S*(*G*)∩*M*)⋊*M*/(*S*(*G*)∩*M*). Let $\overset{-}{G}=G/(S(G)\cap M),\overset{-}{S}(G)=S(G)/(S(G)\cap M)$, and $\overset{-}{M}=M/(S(G)\cap M)$. By N/C-theorem, $N_{\overset{-}{G}}(\overset{-}{S}(G))/C_{\overset{-}{G}}(\overset{-}{S}(G))≲\text{Aut}(\overset{-}{S}(G))$. That is, $\overset{-}{G}/C_{\overset{-}{G}}(\overset{-}{S}(G))=\overset{-}{S}(G)\overset{-}{M}/C_{\overset{-}{G}}(\overset{-}{S}(G)))≲\text{Aut}(\overset{-}{S}(G))$. Note that $\text{ Aut}(\overset{-}{S}(G))$ is abelian since $\overset{-}{S}(G)$ is cyclic. Moreover, $\overset{-}{M}≅S(G)M/S(G)=G/S(G)$ is a nonabelian simple group and $\overset{-}{S}(G)\overset{-}{M}/C_{\overset{-}{G}}(\overset{-}{S}(G))≅(\overset{-}{S}(G)\overset{-}{M}/\overset{-}{S}(G))/(C_{\overset{-}{G}}(\overset{-}{S}(G))/\overset{-}{S}(G))$. Here $\overset{-}{S}(G)\overset{-}{M}/\overset{-}{S}(G)≅\overset{-}{M}$. Therefore, one has $C_{\overset{-}{G}}(\overset{-}{S}(G))/\overset{-}{S}(G)=1$ or $C_{\overset{-}{G}}(\overset{-}{S}(G))/\overset{-}{S}(G)=\overset{-}{S}(G)\overset{-}{M}/\overset{-}{S}(G)=\overset{-}{G}/\overset{-}{S}(G)$. If $C_{\overset{-}{G}}(\overset{-}{S}(G))/\overset{-}{S}(G)=1$, it follows that $\overset{-}{S}(G)\overset{-}{M}/\overset{-}{S}(G)≲\text{Aut}(\overset{-}{S}(G))$ is abelian, a contradiction. If $C_{\overset{-}{G}}(\overset{-}{S}(G))/\overset{-}{S}(G)=\overset{-}{G}/\overset{-}{S}(G)$, then $\overset{-}{S}(G)\leq Z(\overset{-}{G})$. It follows that $\overset{-}{G}=\overset{-}{S}(G)\times \overset{-}{M}$ and then *M*⊴*G*; this contradicts that all maximal subgroups of *G* are nonnormal. Thus, our assumption is not true, so Φ(*G*) = *S*(*G*).

It follows that *G*/Φ(*G*)≅*PSL*(2,5) or *PSL*(2,13).

If Φ(*G*) = 1, then *G*≅*PSL*(2,5) or *PSL*(2,13).

Next, suppose that Φ(*G*) ≠ 1. Let *p* be any prime divisor of |Φ(*G*)|. We claim that *p*≯2. Otherwise, assume that *p* > 2. Let *T* be a subgroup of Φ(*G*) such that Φ(*G*)/*T*≅*ℤ*_*p*_. That is, Φ(*G*/*T*)≅*ℤ*_*p*_. Then (*G*/*T*)/*ℤ*_*p*_≅(*G*/*T*)/Φ(*G*/*T*) = (*G*/*T*)/(Φ(*G*)/*T*)≅*G*/Φ(*G*)≅*PSL*(2,5) or *PSL*(2,13). Since *p* > 2 and Schur multipliers of both *PSL*(2,5) and *PSL*(2,13) are *ℤ*_2_, we have that *G*/*T*≅*PSL*(2,5) × *ℤ*_*p*_ or *PSL*(2,13) × *ℤ*_*p*_. Note that |*𝒩𝒞*(*PSL*(2,5) × *ℤ*_*p*_)|>3 and |*𝒩𝒞*(*PSL*(2,13) × *ℤ*_*p*_)|>3. It follows that |*𝒩𝒞*(*G*)|>3, a contradiction. Thus, *p*≯2, so Φ(*G*) is a cyclic 2-group. If |Φ(*G*)| = 2^*n*^ > 2, let *L* be a subgroup of Φ(*G*) such that Φ(*G*)/*L*≅*ℤ*_2_. Then (*G*/*L*)/*ℤ*_2_≅(*G*/*L*)/Φ(*G*/*L*) = (*G*/*L*)/(Φ(*G*)/*L*)≅*G*/Φ(*G*)≅*PSL*(2,5) or *PSL*(2,13). We have that *G*/*L*≅*SL*(2,5) or *SL*(2,13). Let *M* be a subgroup of *L* such that *L*/*M*≅*ℤ*_2_. Then (*G*/*M*)/*ℤ*_2_≅(*G*/*M*)/(*L*/*M*)≅*G*/*L*≅*SL*(2,5) or *SL*(2,13). Since Schur multipliers of both *SL*(2,5) and *SL*(2,13) are trivial, we have that *G*/*M*≅*SL*(2,5) × *ℤ*_2_ or *SL*(2,13) × *ℤ*_2_; this contradicts that all maximal subgroups of *G* are nonnormal. Thus, |Φ(*G*)| = 2. It follows that *G*≅*SL*(2,5) or *SL*(2,13).

Lemmas 10 and 11 combined together give Theorem 1. 
